# Bioactive Peptides and Exercise Modulate the AMPK/SIRT1/PGC-1α/FOXO3 Pathway as a Therapeutic Approach for Hypertensive Rats

**DOI:** 10.3390/ph15070819

**Published:** 2022-07-01

**Authors:** Jou-Hsuan Ho, Rathinasamy Baskaran, Ming-Fu Wang, Hong-Siang Yang, Yun-Hsin Lo, Zuhair M. Mohammedsaleh, Wan-Teng Lin

**Affiliations:** 1 Department of Food Science, Tunghai University, Taichung 407705, Taiwan; jhho@thu.edu.tw (J.-H.H.); g09621013@thu.edu.tw (H.-S.Y.); 2 Department of Bioinformatics and Medical Engineering, Asia University, Taichung 413305, Taiwan; baskaran@asia.edu.tw; 3 Department of Food and Nutrition, Providence University, Taichung 43301, Taiwan; mfwang@pu.edu.tw; 4 Department of Hospitality Management, College of Agriculture, Tunghai University, Taichung 407705, Taiwan; g10667004@thu.edu.tw; 5 Department of Medical Laboratory Technology, Faculty of Applied Medical Sciences, University of Tabuk, Tabuk 71491, Saudi Arabia; zsaleh@ut.edu.sa

**Keywords:** bioactive peptide, exercise, AMPK, hypertension, mitochondrial biogenesis

## Abstract

Peptides are fragments of fundamental protein sequences that may have health benefits in addition to basic dietary benefits. Recently, we have reported on the pharmacological benefits of alcalase potato protein hydrolysate (APPH) and bioactive peptides isolated from APPH. The aim was to evaluate the synergistic effect of exercise along with DIKTNKPVIF (DF) peptides in ameliorating hypertension in spontaneously hypertensive rat (SHR) rats. We examined ECG parameters, lipid profiles, cardiac markers, and histology, and quantified the proteins associated with fibrosis, hypertrophy, apoptosis, mitochondrial biogenesis, and longevity pathways. DF peptide administration, along with exercise, reduced the blood pressure and cardiac marker levels in serum. Furthermore, it also suppressed the expression of fibrosis markers COL1A1, CTGF, and uPA and downregulated cardiac-hypertrophy-associated markers such as calcineurin, NFATC3, GATA4, pGATA4 and BNP. Exercise synergistically increases the expression of IFG1, PI3K, and AKT cell-survival pathway proteins, along with DF administration. Moreover, AMPK/SIRT1/PGC-1α/FOXO3 pathway protein expression was increased with the combinatorial administration of DF and exercise. Our data suggest that exercise, along with DF peptides, act synergistically in alleviating hypertension by activating the mitochondrial biogenesis pathway.

## 1. Introduction

One of the most prevalent cardiovascular disorders is hypertension. Chronic hypertension raises blood pressure and volume burden in the heart [1]. The increasing pressure load during the contraction period puts extra strain on the ventricular and muscular membranes, causing myocardial hypertrophy [2]. Once collagen deposits are over 20%, this could result in fibrosis, which will cause heart relaxation and contraction dysfunction and heart failure [3]. The pathological development of cardiac hypertrophy, fibrosis, and heart failure could injure myocardial tissue. Thus, determining the therapy for treating hypertension in cardiac muscle damage and understanding its process is critical [4].

Mitochondria is the primary source of energy in all eukaryotic cells, and they are abundant in organs such as the heart, which are in need of high energy to function. Mitochondrial remodeling activities, including biogenesis and repair, dynamics, and mitophagy, all contribute to mitochondrial function [5]. Mitochondrial dysfunction, which is frequent under this condition, could occur if this pathway is disrupted. There is a strong relationship between workload and energy production needs, and myocardial hypertrophy will certainly induce abnormalities in mitochondrial function. Myocardial hypertrophy, fibrosis, and hypertension have all been linked to mitochondrial dysfunction [6].

Insulin-like growth factor 1 (IGF1) has been reported to prevent mitochondrial dysfunction and restore normal mitochondrial function [7]. IGF-1 prevents myocardial injury and, under hypertension, IGF1 levels were decreased in the cardiomyocytes [8]. These effects are mediated by insulin-like growth factor 1 receptors (IGF1Rs), which are distributed throughout the heart. IGF1R signaling cascade triggers cell survival by activating phosphatidylinositol 3-kinase (PI3K) and protein kinase B (AKT) [9]. For the B-cell lymphoma 2 (BCL-2) family proteins, phosphorylated AKT serves as a pro-survival pathway. Within the BCL-2 family, there are pro-apoptotic and pro-survival subgroups. Bcl-2 and B-cell lymphoma-extra-large (Bcl-xL) are pro-survival proteins that prevent the activation of downstream apoptotic signals [10].

Previous research has found that oxidative stress, apoptosis, and fibrosis of cardiomyocytes are all linked to mitochondrial dysfunction during hypertension [11,12,13]. Sirtuin 1 (SIRT1) is one of the key redox-sensitive enzyme-controlling mitochondrial biogenesis and regulates several cellular events, such as apoptosis, cell survival, endocrine signaling, and chromatin remodeling [14]. SIRT1 activation stimulates mitochondrial biogenesis through deacetylation and transcriptional activation of the Peroxisome proliferator-activated receptor gamma coactivator 1-alpha (PGC-1α). AMP-activated protein kinase (AMPK) is another key regulatory protein regulating mitochondrial biogenesis along with SIRT1, and they are both reported to be evolutionarily conserved partners [15]. Forkhead box O3 (FOXO3) is one of the downstream regulators of SIRT1; the activation of FOXO3 through SIRT1 protects the cells from oxidative stress and prevents cell death. the up-regulation of SIRT1 in the heart tissue protects the heart from hypertrophy and myocardial infarction [16]. Cardiac hypertrophy is characterized by cardiomyocyte enlargement and occurs in response to mechanical stress or other stimuli. Alterations in mitochondrial biogenesis accompany pathological hypertrophy [17]. In rats, hypertrophy is inhibited through H_2_S and/or mitochondrial biogenesis pathway [18].

Due to their antioxidant properties, biologically active peptides have attained a significant amount of interest, and they have a well-understood mechanism that involves hydrogen atom transfer, single electron transfer, and chelating transition pro-oxidant metals [19]. Bioactive peptides possess anti-oxidant properties by inhibiting lipid peroxidation and reactive oxygen species (ROS) generation; they also increase cellular anti-oxidant enzymes, such as superoxide dismutase, glutathione peroxidase, and catalase. Peptide from potato hydrolysate induces several antioxidant enzymes in the SHR rat kidney through activating NRF2 [20]. Angiotensin-converting enzyme (ACE) inhibitor peptides can be found in foods rich in proteins, such as casein, zein, soybean protein, dried salted fish, ovalbumin, fish, and sauce [21]. Regularly eating foods that have active peptides with ACE-inhibiting activity could control blood pressure naturally [22]. Functional nutritious foods include proteins, protein hydrolyzes, and peptides generated from hydrolyzed dietary proteins and fermentation products. Previous studies have demonstrated that peptides produced from plants such as soybean and potato have anti-hypertension, anti-cancer, hypocholesterolemic, and anti-obesity properties [23]. The bioactive peptides extracted from soybeans also lower risk factors linked to cardiovascular disease [24]. Potato protein hydrolyzate (PPH) has been found to protect the intestinal mucosa from ethanol-induced damage due to its antioxidant action [25]. Our previous research suggests that APPH protects the liver from lipid buildup, apoptosis, and fibrosis caused by a high-fat diet [26]. The DIKTNKPVIF peptide isolated from potato hydrolysates also helps to avoid hepatic steatosis [27].

In cardiac rehabilitation, regular exercise has been shown to be beneficial in the prevention and treatment of cardiovascular disease [28]. Aerobic exercise has been shown to improve cardiovascular adaptability during hypertension in clinical and experimental trials [29]. Recently, we have reported that the DF peptide could prevent fibrosis and hypertrophy in hypertensive rats [30]. However, the effect of DF administration and exercise on cardiac apoptosis and cardiac mitochondria biogenesis in hypertension is unclear. In the current study, we hypothesized that exercise and DF administration might ameliorate hypertension in the SHR model and, if so, the anti-hypertensive effects of DF and exercise may be partly due to the regulation of the AMPK/SIRT1/PGC-1α/FOXO3 pathway.

## 2. Results

### 2.1. Effect of DF Bioactive Peptide and a Combination of Exercise (EX) on Whole Heart Weight, Blood Pressure, and Lipid Profile of WKY and SHR Rats

Animals were separated into five groups after an acclimatization period; SHR rats were randomly divided into treatment groups: Group I—WKY rats, Group II—SHR, Group III—SHR + EX, Group IV—SHR + DF, and Group V—SHR + DF + EX.

**Figure 1:**
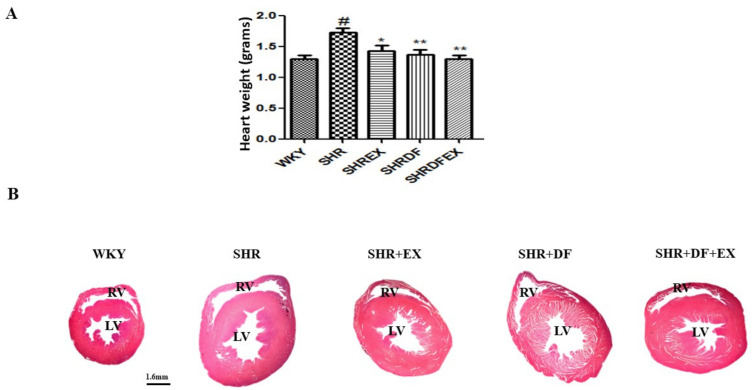
Effect of DF bioactive peptide and combination of exercise on SHR rat heart. (**A**) heart weight (**B**) tomography of cardiac tissue. Each bar represents mean ± SD (*n* = 8). # *p* < 0.01 compared to WKY, * *p* < 0.05 and ** *p* < 0.01 compared to SHR.

**Figure 2:**
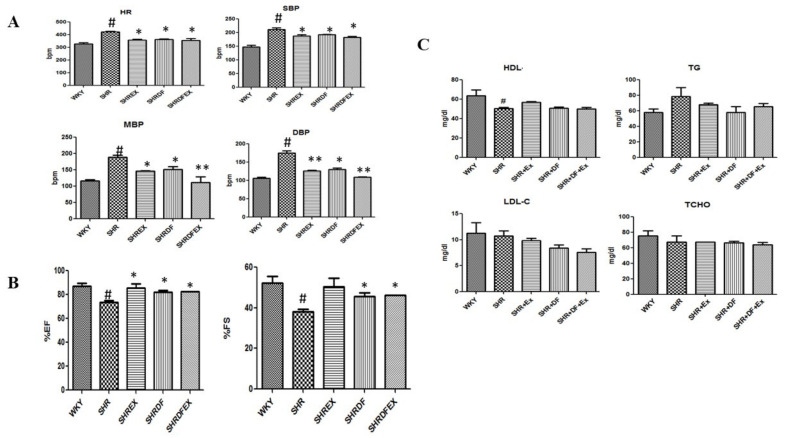
Effect of DF bioactive peptides and exercise on hemodynamic parameters, and lipid profile of WKY and SHR rats. (**A**) Heart rate and Blood pressure. (**B**) Ejection fraction (EF) and Fractional shortening (FS). (**C**). Lipid profile. HR—Heart rate, SBP—systolic blood pressure, MBP—mean blood pressure, DBP—diastolic blood pressure, HDL—high density lipoprotein, TG—Triglycerides, LDL-C—low density lipoprotein cholesterol, TCHO—Total cholesterol. Each bar represents mean ± SD (*n* = 8). (*n* = 8) # *p* < 0.01 compared to WKY, * *p* < 0.05 and ** *p* < 0.01 compared to SHR.

Increased heart volume and blood pressure are characteristic features of hypertrophy and hypertension. In our present study, the heart weights of SHR rats increased compared to WKY rats. The heart weights of SHR rats treated with DF peptide and normalized with a combination of exercise decreased (Figure 1). DF bioactive peptide and a combination of exercise normalized the blood pressure of hypertensive rats when compared to SHR rats. Furthermore, DF bioactive peptide and exercise normalized the percentage of ejection fraction and fractional shortening when compared to SHR rats. However, no significant changes were observed in the serum lipid profile of WKY and SHR rats (Figure 2).

### 2.2. Effect of DF and Exercise on Myocardial Injury Markers and Cardiac, Hepatic, and Renal Function Markers of WKY and SHR Rats

**Figure 3:**
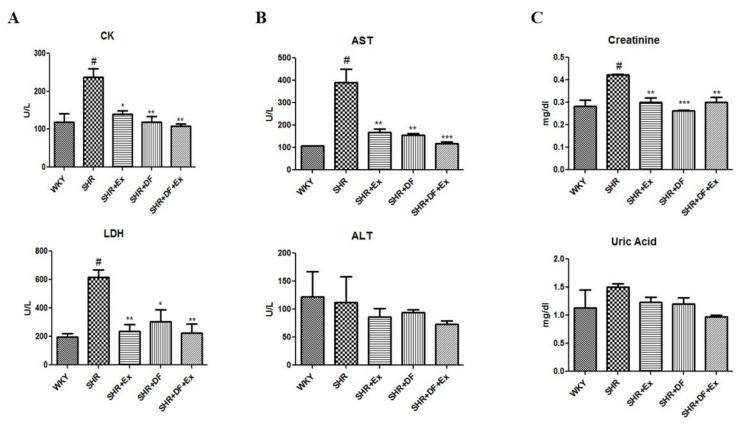
Effect of DF and exercise on myocardial injury markers and cardiac, hepatic, and renal function markers of WKY and SHR rats. (**A**) Creatine kinase and lactate dehydrogenase. (**B**) Aspartate transaminase and alanine transaminase. (**C**). Creatinine and uric acid. Each bar represents mean ± SD (*n* = 8). # *p* < 0.01 compared to WKY, * *p* < 0.05, ** *p* < 0.01 and *** *p* < 0.001 compared to SHR.

Serum parameters such as creatine kinase (CK), lactate dehydrogenase activity (LDH), uric acid, creatine, aspartate transaminase, alanine transaminase, and creatine kinase parameters are summarized in Figure 3. Myocardial injury is accompanied by an increase in CK and LDH. In the SHR group, the activities of serum CK and LDH were significantly increased when compared to the WKY group. DF treatment and exercise alone and combined significantly reduced the CK and LDH activity in SHR rats. Additionally, hepatic marker aspartate aminotransferase (AST) and renal marker creatinine were significantly increased in the SHR group compared to the WKY group. DF treatment and exercise in SHR rats significantly reduced the AST and creatinine levels when compared to the SHR alone group. However, other hepatic and renal markers, alanine transaminase (ALT), and uric acid were not altered in the SHR group when compared to the WKY control group.

### 2.3. Effects of DF Peptide and Exercise on Cardiac Structure in Rats

**Figure 4:**
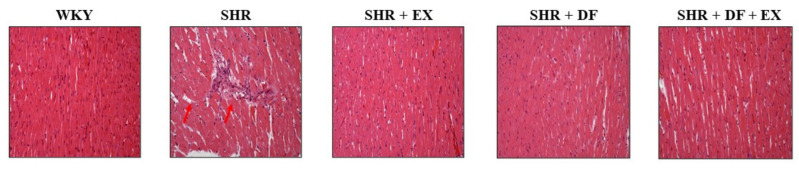
Effects of DF peptide and exercise on cardiac structure in rats. H&E staining.

HE staining was used to detect the pathological changes in rats’ cardiac structure. The results showed that, compared with WKY rats, cardiomyocytes in SHR rats had blurred cell edges and deformations in the cell structure. DF treatment and exercise ameliorated myocardial injury in SHR rats. Combined DF treatment and exercise in the SHR rats effectively reduced the myocardial abnormalities (Figure 4).

### 2.4. DF Peptide Administration and Exercise Inhibits Apoptosis in SHR Heart

**Figure 5:**
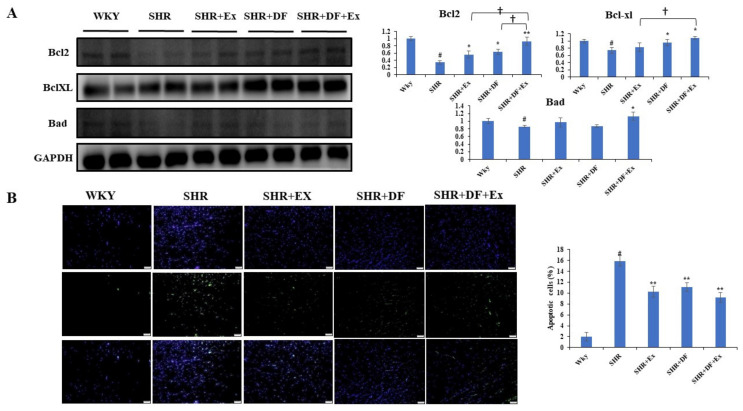
DF peptide administration and exercise inhibits apoptosis in SHR heart. (**A**) Western blot of apoptosis protein markers. (**B**) TUNEL assay. Each bar represents mean ± SD (*n* = 8). # *p* < 0.01 compared to WKY, * *p* < 0.05 and ** *p* < 0.01 compared to SHR, † *p* < 0.05 compared to SHR + EX and/or SHR + DF.

The effect of DF peptide administration and exercise in apoptosis in SHR rat heart was evaluated. Figure 5A shows the expression levels of apoptotic pathway proteins in different treatment groups by Western blot. DF peptide administration and exercise in combination increased the expression of anti-apoptotic proteins Bcl2 and Bcl-XL and reduced the expression of pro-apoptotic Bad expression in SHR rats. A terminal deoxynucleotidyl transferase dUTP nick end labeling (TUNEL) staining assay was performed to determine the efficacy of DF peptide administration and exercise in SHR rats. In the SHR group, the number of TUNEL-positive cells was significantly increased compared to the control WKY group. Both DF and exercise effectively decreased the TUNEL-positive cells compared to the SHR group (Figure 5B).

### 2.5. Effects of DF Peptide Treatment and Exercise in Cardiac Fibrosis

**Figure 6:**
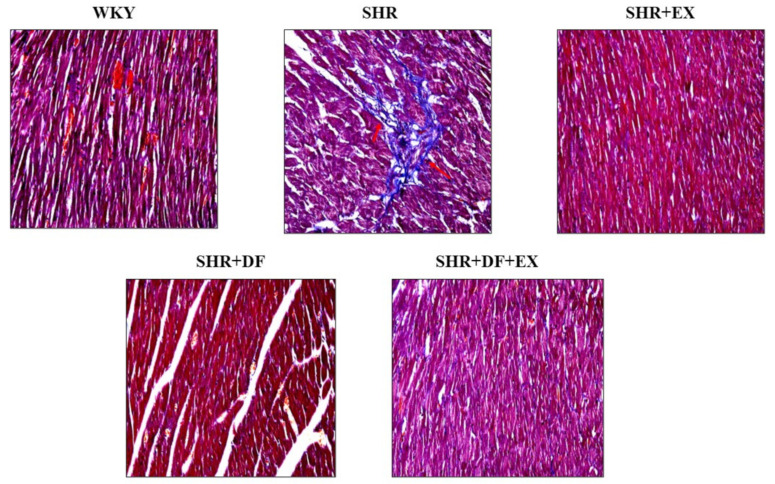
Effects of DF peptide treatment and exercise in cardiac fibrosis. Masson Trichrome Staining.

Figure 6 shows the transverse section of the heart tissue stained with Masson’s trichrome in the different treatment groups. The WKY group showed a normal heart section, whereas, in the SHR group, dark-blue staining was observed, which indicated the presence of collagen accumulation, which is a hallmark of fibrosis. Dark-blue staining was reduced in the SHR rats, which received DF treatment and exercise both alone and combined.

### 2.6. Effect of DF and Exercise on the Fibrosis-Associated Proteins in the Heart Tissue of SHR Animals

**Figure 7:**
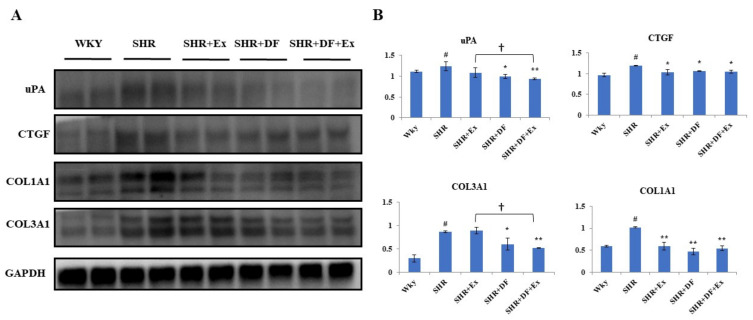
Effect of DF and exercise on the fibrosis-associated proteins in the heart tissue of SHR animals. (**A**) Expression of collagen type I α 1 chain, (COL1A1), COL3A1 CTGF, and tissue-type plasminogen activator protein uPA proteins. (**B**) Graph showing densitometric analysis. Each bar represents mean ± SD (*n* = 8). # *p* < 0.01 compared to WKY, * *p* < 0.05 and ** *p* < 0.01 compared to SHR, † *p* < 0.05 compared to SHR + EX.

Western blot assay was employed to detect fibrotic protein markers such as urokinase plasminogen activator (uPA), connective tissue growth factor (CTGF), collagen-type I α 1 chain (COL1A1), and collagen-type III α 1 chain (COL3A1) in all the treatment groups (Figure 7). The protein expression of uPA, CTGF, COL1A1, and COL3A1 in the SHR group was found to be significantly increased compared to the WKY control group. DF treatment and exercise alone significantly reduced the expression of these fibrosis-associated proteins. However, combined DF treatment and exercise have a better effect on inhibiting the fibrosis-associated proteins uPA and COL1A1 in SHR rats.

### 2.7. Effect of DF and Exercise on Cardiac Hypertrophy Markers

**Figure 8:**
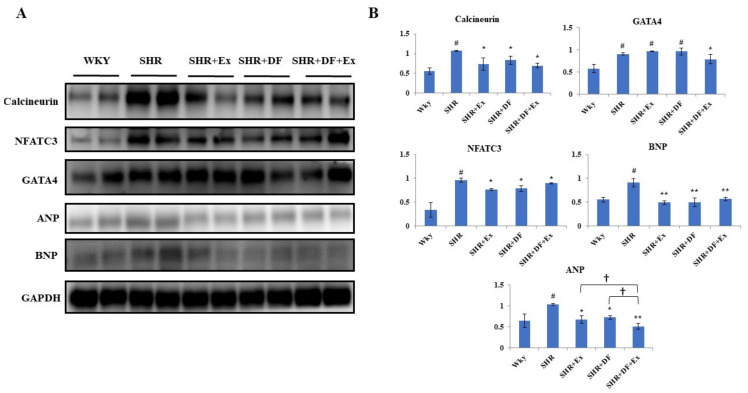
Effect of DF and exercise on cardiac hypertrophy markers. (**A**) Expression of cardiac hypertrophy-associated markers such as calcineurin, NFATC3, GATA4, ANP, and BNP proteins. (**B**) Graph showing densitometric analysis. Each bar represents mean ± SD (*n* = 8). # *p* < 0.01 compared to WKY, * *p* < 0.05 and ** *p* < 0.01 compared to SHR, † *p* < 0.05 compared to SHR + EX and/or SHR + DF.

Hypertrophy plays an important role in SHR rats; therefore, the expression levels of hypertrophy markers such as calcineurin, nuclear factor of activated T cells 3 (NFATC3), GATA-binding protein 4 (GATA4), brain natriuretic peptide (BNP), and atrial natriuretic peptide (ANP) were evaluated in all the treatment groups (Figure 8). Western blot results revealed that the protein expression of calcineurin, NFATC3, GATA4, BNP, and ANP were significantly increased in the heart tissue of the SHR group compared to the control WKY group. DF and exercise treatment alone in SHR rats significantly inhibited the hypertrophy protein markers. However, DF peptide treatment and exercise combined synergistically reduced the expression levels of cardiac hypertrophy marker ANP.

### 2.8. Effect of Bioactive Peptide and Exercise on Survival Protein (PI3K/AKT) Markers and IGF1 Activation

**Figure 9:**
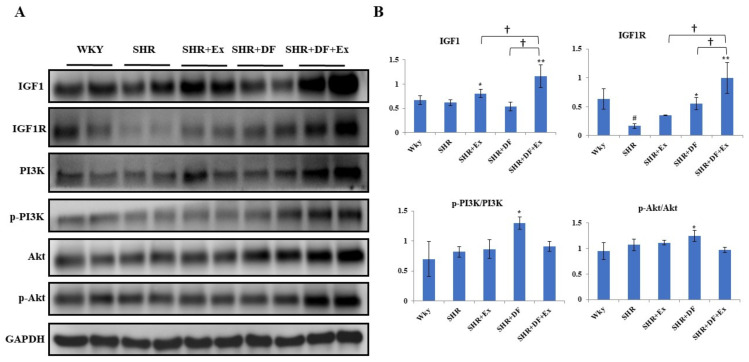
Effect of bioactive peptide and exercise on survival protein (PI3K/AKT) markers and IGF1 activation. (**A**) Expression of survival protein PI3K/AKT and IGF1. (**B**) Graph showing densitometric analysis. Each bar represents mean ± SD (*n* = 8). # *p* < 0.01 compared to WKY, * *p* < 0.05 and ** *p* < 0.01 compared to SHR, † *p* < 0.05 compared to SHR + EX and/or SHR + DF.

Next, the protein expression levels of survival proteins were observed (Figure 9). In SHR rats, there were no significant changes in the expression of IGF1, PI3K, or AKT, but IGF1R expression was significantly reduced compared to the control WKY group. Exercise increased IGF1 levels, and DF treatment significantly increased IGF1R levels. However, both DF and exercise significantly increased both IGF1 and IGF1R compared to the SHR, DF, and exercise groups. DF treatment significantly increased the p-PI3K and p-AKT levels. But exercise alone, and combined with DF, had no effect on PI3K and AKT activation.

### 2.9. DF Treatment and Exercise Activates Mitochondrial Biogenesis through Activation of AMPK/SIRT1/PGC-1α/FOXO3 Pathway

**Figure 10:**
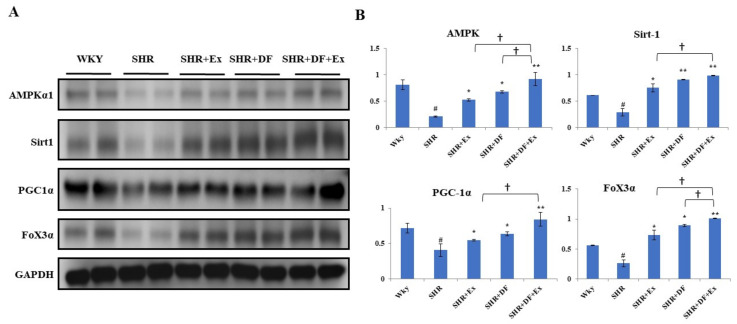
DF treatment and exercise activate mitochondrial biogenesis through the activation of the AMPK/SIRT1/PGC-1α/FOXO3 pathway. (**A**) Expression of AMPKα1, Sirt1, PGC1α, and FoX3a proteins. (**B**) Graph showing densitometric analysis. Each bar represents mean ± SD (*n* = 8). # *p* < 0.01 compared to WKY, * *p* < 0.05 and ** *p* < 0.01 compared to SHR, † *p*< 0.05 compared to SHR + EX and/or SHR + DF.

We evaluated the role of AMPK and its downstream regulators, which are involved in mitochondrial biogenesis, SIRT1/PGC-1α/FOXO3 (Figure 10). In the SHR group, the expression levels of AMPK, SIRT1, PGC-1α, and FOXO3 were significantly reduced compared to the WKY group. Exercise and administration of the DF peptide alone in SHR animals significantly increased AMP-activated protein kinase (AMPK), SIRT1, PGC-1α, and FOXO3 expression. However, combining exercise and DF treatment led to higher significance than DF and exercise alone, suggesting its synergistic effect in inducing the expression of AMPK, SIRT1, PGC-1α, and FOXO3 in SHR animals.

## 3. Discussion

In this study, we identified that the pure peptide amino acid sequence DIKTNKPVIF and exercise attenuated hypertension by inhibiting fibrosis, hypertrophy, and myocardial apoptosis and activating mitochondrial biogenesis pathway protein expression in spontaneously hypertensive rats.

One of the decisive factors in cardiovascular disorder is hypertension [31]. Pressure overload causes ventricular hypertrophy and fibrosis by imposing mechanical stress on the ventricles [32]. Bioactive peptides have recently been shown to play an important role in preventing the advancement of heart disease [33]. Exercise training has been shown to provide multiple benefits, such as anti-hypertension, anti-diabetes, anti-obesity, and anti-lipidemia [34]. In SHR animals, we detected a significant increase in hepatic serum aminotransferases and renal functional markers such as serum uric acid and creatine levels, in addition to myocardial dysfunction. Congestion and liver necrosis are caused by right ventricular failure and inadequate cardiac output [35]. In previous studies, heart failure has been linked to chronic kidney disease and a reduction in renal function [36]. The SHR group demonstrated structural alterations in heart weight as well as the heart weight–body weight ratio. In addition, compared to WKY rats, pathological abnormalities in the heart of SHR rats indicate an increased myocyte area and collagen deposition. Regarding the structural and pathological alterations in SHR, the current findings are congruent with those of Iliev et al. [37]. Treatment with bioactive peptide DF and exercise alone significantly enhanced cardiac function, while also acting as a preventive element against kidney and liver impairment in hypertensive rats. Our previous report showed that peptides isolated from soybean (VHVV) and potato (DIKTNKPVIF) both reduce the development of high-fat-diet-induced liver steatosis [27,38]. Exercise training is becoming a viable nonpharmacological therapy option for cardiovascular disorders. Regular exercise training has been shown to improve cardiac enzyme expression and activity in several studies [34,39]. Exercise training was found to provide significant cardioprotection against myocardial ischemia by reducing mitochondrial damage [40].

H-FABP rapidly diffused into the plasma following SHR myocardial damage, suggesting that it might be employed as a measure of early myocardial injury [41]. Cardiac fibroblast proliferation and phenotypic plasticity are essential mediators in myocardial damage remodeling [42]. Myofibroblasts produce CTGF, which is a reliable biomarker of the myofibroblast phenotype when paired with the development of stress fibers [43]. ANP has proven to be the most effective marker for identifying heart failure. In clinical studies, the elevated plasma levels of ANP and BNP are directly correlated with the severity of cardiac dysfunction [44]. ANP and BNP levels were also found to be high in patients with a variety of cardiovascular disorders [45]. In our present study, DF treatment and exercise significantly reduced the expression of fibrotic protein markers such as uPA, CTGF, COL1A1, and COL3A1, as well as hypertrophy markers such as NFATC3, GATA4, BNP, and ANP, which indicates that it could attenuate hypertension-mediated myocardial injury.

Apoptosis plays a critical role in cardiomyocyte cell death during hypertension. Several studies have shown that cardiovascular diseases such as hypertension could induce apoptosis in the myocardial cell [46,47]. In our present study, the SHR group had more apoptotic cells in the TUNEL assay, which were reduced after combined DF treatment and exercise. Further, we looked at apoptotic protein indicators to study the molecular level. The DF bioactive peptide and exercise increased the expression of antiapoptotic on markers (Bcl-2/Bcl-XL), resulting in cardiac survival. Hypertension has been shown to alter the PI3K/Akt cell survival pathway in cardiomyocytes [48]. During exercise in rats, the PI3K-Akt survival pathway may be engaged, acting as a compensatory strategy [49]. The PI3K/Akt cell survival pathways have been shown to be important in preserving the heart from STZ-induced diabetes [50]. The expression of IGF1R was reduced in the SHR group. The activation of the IGF1R-PI3K-Akt signaling cascade by APPH protects against cardiomyocyte loss in rats [51]. In this study, combined DF administration and exercise increased the expression of IGF1R compared to DF and exercise alone, implying changes in cardiomyocyte survival.

The activation of AMPK is triggered by an increase in the intracellular AMP/ATP ratio. AMP-activated protein kinase (AMPK) is key in the regulation of energy metabolism. AMPK regulates the balance of energy generation and consumption in the body to maintain energy homeostasis [52]. AMPK increases SirT1 activity, which can lead to deacetylation of the transcription factor FOXO3, which regulates energy metabolism [53]. By raising the ratio of Bcl-2/BAX, the AMPK signaling pathway could possibly have anti-apoptotic effects [54]. Our results showed that DF administration and exercise could activate the AMPK/SirT1/ PGC-1α/FOXO3 pathway. AMPK inhibitors have previously been shown to decrease the activation of the AMPK/SirT1/FOXO3 pathway in H9c2 cardiomyocyte and accelerate apoptosis [51]. A previous study by Campos et al. found that an 8-week exercise program restored the pool of healthy mitochondria in post-MI failing hearts and corrected the substantial myocardial infraction-induced rise in the mitochondrial number–size ratio, indicating an accumulation of smaller and fragmented mitochondria [55]. DF and exercise may inhibit myocardial apoptosis by regulating the energy metabolism pathway (AMPK/SirT1/ PGC-1α/FOXO3).

In conclusion, DF peptides from potato and exercise could be used to treat myocardial injury caused by hypertension. DF peptides and exercise act synergistically and attenuate the myocardial injury induced by spontaneous hypertension, possibly by regulating the energy metabolism signaling pathway (AMPK/SirT1/PGC-1α/FOXO3), as well as preventing myocardial fibrosis, hypertrophy, and apoptosis.

## 4. Materials and Methods

### 4.1. Animal Procedure

BioLasco Co., Ltd. (Taipei, Taiwan) provided 12-week-old SHR and Wistar Kyoto (WKY) rats. All the rats were fed regular rat feed and given tap water, and they were kept at a constant temperature (22 °C) with a 12-h light/dark cycle. Animals were separated into five groups after the acclimatization period; SHR rats were randomly divided into treatment groups (*n* = 8): Group I–WKY rats, Group II–SHR, Group III–SHR + EX, Group IV–SHR + DF, and Group V–SHR + DF + EX. The therapy lasted approximately eight weeks. The animals were slaughtered after treatment. Blood was collected and serum was separated. Hearts were dissected from the animals and washed with ice-cold phosphate-buffered saline (PBS). Both the serum and heart were stored at −80 °C until further analysis.

### 4.2. Drug Treatment

Bioactive decapeptide DF (DIKTNKPVIF (D—aspartic acid; I—isoleucine; K—lysine; T—threonine; N—asparagine; K—lysine; P—proline; V—valine; I—isoleucine; F—phenylalanine)) was purchased commercially from DG peptides Co. Ltd., Hangzhou, China, and was of 98% purity. DF dissolved in PBS at 10 mg/kg BW was administered to the SHR rats for 8 weeks through intragastric injection. The control group received PBS through intragastric injection. For exercise training, animals were allowed to swim freely. Animals were trained to swim for 20, 40, and 60 min at the 1st, 2nd, and 3rd week, respectively; from the 4th to the 8th week, the animals were allowed to swim for 60 min.

### 4.3. Blood Pressure Measurement

The primary hemodynamic parameters such as heart rate, systolic blood pressure, mean blood pressure, and diastolic blood pressure, were analyzed in the treatment groups using a noninvasive blood pressure measurement system (Softron, BP-2010 series).

### 4.4. Measurement of Blood Serum Biochemical Parameters

Blood biochemical analysis for lipid profile and organ-specific markers were quantified using specific ELISA kits (Sigma Aldrich, St. Louis, MO, USA). Biochemical analysis was performed based on the protocol provided by the kit manufacturer.

### 4.5. Tissue Staining

Heart tissue stored in 10% formalin solution was washed and covered with paraffin wax. Using microtome, paraffin-embedded heart tissue was sequentially sectioned at 2-µm thickness. Tissue sections were placed in glass slides and processed for staining. For hematoxylin and eosin (H&E) staining, glass slides with heart tissue sections were deparaffinized in a hot air oven and stained with H&E; excess stains were washed with PBS and dehydrated with graded alcohols (100%, 95%, and 75%). Finally, the slides were observed, and images were captured using a microscope (Carl Zeiss Microscopy, Thornwood, NY, USA). For masson trichome staining, the glass slides with tissue sections were stained with masson trichome stain; with H&E, excess stains were washed with PBS and dehydrated with graded alcohols (100%, 95%, and 75%). The slides were observed, and images were captured using a microscope (Carl Zeiss Microscopy, Thornwood, NY, USA). For TUNEL assay, a TUNEL kit (Roche Applied Science, Indianapolis, IN, USA) was used according to the manufacturer’s protocol.

### 4.6. Tissue Protein Extraction and Western Blotting

Western blotting was performed following previously reported methods [56]. A total of 100 mg of heart tissue was washed with ice-cold PBS and homogenized in a lysis buffer (CelLytic^TM^ MT Cell Lysis Reagent, Sigma-Aldrich, Saint Louis, MO, USA) with a protease-inhibitor cocktail (PIC). Homogenate was then centrifuged at 12,000 RPM for 20 min at 4 °C. After centrifugation, the supernatant was collected and stored at −80 °C in aliquots until Western blot analysis. The protein concentrations in each sample were quantified by Lowry’s method, and an equal amount (30–40 µg) of protein was separated by using an 8–12% SDS-PAGE electrophoresis gel at 90 V for 45 min. Protein from the gel was transferred to the PVDF membrane at 4 °C using blotting apparatus (Bio-Rad Laboratories, Hercules, CA, USA). The PVDF membrane was then submerged in 5% non-fat milk powder in TBST at room temperature for 1 h. The membrane was then washed with TBS three times, for 5 min each. The membrane was then incubated overnight with primary antibody (1:1000 dilution in TBST) at 4 °C in a mechanical rocker. Then, the membrane was washed with TBS three times, for 5 min each, and incubated with HRP-conjugated secondary antibody (1:5000 dilution in TBST) at room temperature for 1 h in a mechanical rocker. After washing with TBS, the membrane was submerged in chemiluminescence ECL solution (Merck Millipore, Burlington, MA, USA), the protein bands were visualized using chemi-doc apparatus (Fujifilm LAS-3000, GE Healthcare), and densitometric analysis was performed using ImageJ software (version 1.4.3.67) (NIH, Bethesda, MD, USA)

The antibodies used in the present study and their manufacturer details are as follows: AKT (SC-5298, Santa Cruz Biotechnology, Santa Cruz, CA, USA), p-AKT (#9275s, Cell Signaling, Baltimore, MD, USA), AMPK (SC-19128, Santa Cruz Biotechnology, Santa Cruz, CA, USA), ANP (SC-20158, Santa Cruz Biotechnology, Santa Cruz, CA, USA), Bad (SC-8044, Santa Cruz Biotechnology, Santa Cruz, CA, USA), Bcl2 (SC-7382, Santa Cruz Biotechnology, Santa Cruz, CA, USA), Bcl-xl (SC-8392, Santa Cruz Biotechnology, Santa Cruz, CA, USA), BNP (SC-18818, Santa Cruz Biotechnology, Santa Cruz, CA, USA), Calcineurin (610259, BD Biosciences, San Jose, CA, USA), COL1A1 (SC-28657, Santa Cruz Biotechnology, Santa Cruz, CA, USA), COL3A1 (SC-271249, Santa Cruz Biotechnology, Santa Cruz, CA, USA), CTGF (SC-14939, Santa Cruz Biotechnology, Santa Cruz, CA, USA), FOXO3α (#2497, Cell Signaling, Baltimore, MD, USA), GAPDH (SC-25778, Santa Cruz Biotechnology, Santa Cruz, CA, USA), GATA4 (SC-25310, Santa Cruz Biotechnology, Santa Cruz, CA, USA), IGF1 (SC-9013, Santa Cruz Biotechnology, Santa Cruz, CA, USA), IGF1R (ab39675, Abcam, Cambridge, UK), NFATC3 (SC-1152, Santa Cruz Biotechnology, Santa Cruz, CA, USA), PGC-1α (SC-13067, Santa Cruz Biotechnology, Santa Cruz, CA, USA), PI3K (SC-423, Santa Cruz Biotechnology, Santa Cruz, CA, USA), p-PI3K (SC-12929, Santa Cruz Biotechnology, Santa Cruz, CA, USA), SIRT1 (SC-15404, Santa Cruz Biotechnology, Santa Cruz, CA, USA), and uPA (SC-14019, Santa Cruz Biotechnology, Santa Cruz, CA, USA). The dilution factor for all primary antibodies was 1:1000, and the dilution factor for all secondary antibodies was 1:5000.

### 4.7. Statistical Analysis

All the experiments were performed thrice. The presented data are the mean ± SD. One-way ANOVA with Tukey’s multiple comparisons test was used to compare the statistical difference between the treatment groups. SPSS 16 software (SPSS, Chicago, IL, USA) was used for statistical analysis.

## Data Availability

Data is contained within the article.
